# Mesenchymal Stem Cells Increase Drug Tolerance of A431 Cells Only in 3D Spheroids, Not in 2D Co-Cultures

**DOI:** 10.3390/ijms25084515

**Published:** 2024-04-20

**Authors:** Flóra Vajda, Áron Szepesi, Zsuzsa Erdei, Edit Szabó, György Várady, Dániel Kiss, László Héja, Katalin Német, Gergely Szakács, András Füredi

**Affiliations:** 1 Institute of Molecular Life Sciences, HUN-REN Research Centre for Natural Sciences, 1117 Budapest, Hungary; 2 Doctoral School, Semmelweis University, 1085 Budapest, Hungary; 3 Creative Cell Ltd., 1119 Budapest, Hungary; 4 John von Neumann Faculty of Informatics, Óbuda University, 1034 Budapest, Hungary; 5 Institute of Organic Chemistry, HUN-REN Research Centre for Natural Sciences, 1117 Budapest, Hungary; 6 National Laboratory for Drug Research and Development, 1117 Budapest, Hungary; 7 Center for Cancer Research, Medical University of Vienna, 1090 Wien, Austria; 8 Institute of Technical Physics and Materials Science, HUN-REN Centre for Energy Research, 1121 Budapest, Hungary

**Keywords:** mesenchymal stem cells, cancer cells, tumor microenvironment, drug resistance, spheroids

## Abstract

Mesenchymal stem cells (MSCs) are an integral part of the tumor microenvironment (TME); however, their role is somewhat controversial: conflicting reports suggest that, depending on the stage of tumor development, MSCs can either support or suppress tumor growth and spread. Additionally, the influence of MSCs on drug resistance is also ambiguous. Previously, we showed that, despite MSCs proliferating significantly more slowly than cancer cells, there are chemotherapeutic drugs which proved to be similarly toxic to both cell types. Here we established 2D co-cultures and 3D co-culture spheroids from different ratios of GFP-expressing, adipose tissue-derived MSCs and A431 epidermoid carcinoma cells tagged with mCherry to investigate the effect of MSCs on cancer cell growth, survival, and drug sensitivity. We examined the cytokine secretion profile of mono- and co-cultures, explored the inner structure of the spheroids, applied MSC-(nutlin-3) and cancer cell-targeting (cisplatin) treatments separately, monitored the response with live-cell imaging and identified a new, double-fluorescent cell type emerging from these cultures. In 2D co-cultures, no effect on proliferation or drug sensitivity was observed, regardless of the changes in cytokine secretion induced by the co-culture. Conversely, 3D spheroids developed a unique internal structure consisting of MSCs, which significantly improved cancer cell survival and resilience to treatment, suggesting that physical proximity and cell–cell connections are required for MSCs to considerably affect nearby cancer cells. Our results shed light on MSC–cancer cell interactions and could help design new, better treatment options for tumors.

## 1. Introduction

The tumor microenvironment (TME) is described as a heterogenous, supporting niche that influences tumor cell survival, therapy resistance, and metastasis [1]. The cellular (immune cells, fibroblasts/mesenchymal stem cells (MSCs)), vascular, and non-cellular components (such as extracellular matrix, exosomes, and cytokines) of the TME constantly interact, and therefore alter the gene expression profile of both tumor and stroma cells [2]. Naïve MSCs are mostly located in the adipose tissues and bone marrow, but tumor cells can attract MSCs to establish a supportive milieu [3]. This is a potential mechanism through which tumors might shape their microenvironment by stimulating MSCs to transition from a naïve state to a primed condition resembling cancer-associated fibroblasts (CAFs) or tumor associated-MSCs (TA-MSCs), characterized by the expression of specific markers [4].

MSCs are known to play a dual role in tumor development. MSCs influence their environment through secreted factors including VEGF (vascular endothelial growth factor), FGF-2, PDGF, HGH, BDNF, SDF-1alpha, IGF-1, IGF-2, TGF-beta, TNF-alpha, IGFBP-2, LIF, M-CSF, MIP-2, IL-8, IL-6, and IFN-gamma cytokines, which can increase cancer cell proliferation and survival even in the context of chemotherapy treatment [5]. Cytokines secreted by MSCs, such as IL-6 (interleukin-6), bFGF (basic fibroblast growth factor), and PGE2, drive/accelerate tumor cell growth [5]. VEGF influences vascularization by promoting angiogenesis, thus supplying tumor cells with nutrients and oxygen [5]. On the other hand, naïve MSCs secrete PGE2, lactate, and TGF-beta, which inhibit tumor growth. Cytokines including CCL5, IFN-gamma, and TGF-beta promote EMT, thereby facilitating the invasion and metastasis of epithelial cancer cells. MSCs can also inhibit the AKT and Wnt pathways. MSCs form a structural barrier that hinders the delivery of drugs to tumor cells. Moreover, the presence of MSCs increase c-myc and wnt expression and modify the PI3K/AKT and JAK2/STAT3 signaling pathways, which supports proliferation even after drug treatment [6,7]. Expression of sphingosine-1-phosphate receptor-1 (S1PR1) is also induced in tumors by human bone marrow MSCs, rendering tumors resistant to chemotherapy [8].

Previously, we examined the response of naïve MSCs and cancer cells to different chemotherapeutics [9] and revealed that, despite the clear difference in proliferation rate, MSCs can be susceptible to chemotherapy drugs. Depending on the mechanism of action of the compound, the sensitivity of MSCs may be comparable to that of cell lines isolated from tumors. Here we investigate the role of MSCs in cancer drug resistance by establishing 2D and 3D co-culture systems of GFP-tagged adipose derived MSCs (Ad-MSC-GFP 3) and an mCherry-expressing epidermoid carcinoma cell line (A431-mCh). Three-dimensional multicellular systems offer a more relevant model for drug penetration compared to 2D cell-cultures, potentially leading to more relevant cytotoxicity results. Cellular organization in 3D co-cultures results in a gradient of oxygen, nutrients, and cellular waste. The 3D structures imitate real tumor zone structures that include a proliferative, senescent, and necrotic inner zone [10]. We tested two drugs with distinct mechanisms of action. Cisplatin is one of the most widely used chemotherapeutics in oncology [11], targeting rapidly dividing cancer cells. We previously showed that cisplatin is more toxic to cancer cells such as A431 cells derived from epidermoid carcinoma than to MSCs [9]. Moreover, in epidermoid or squamous cell carcinoma, a large-scale randomized clinical study demonstrated that the combination of cisplatin with radiotherapy increases disease-free survival and reduces both local and distant recurrences [12]. Nutlin-3 is an experimental MDM2-inhibitor which selectively kills cells expressing wild type p53, such as MSCs [13]. 

Our aim was to establish and characterize a tumor model that better reflects physiological conditions, allowing for a more accurate understanding of the interplay between the TME and cancer cells and a more efficient targeting of tumors. In our spheroid model, we monitor the efficacy of various therapies in a coculture of fluorescently labeled MSCs and cancer cells. Based on our results, we conclude that stimulation of cytokine expression, weakening of stromal cells, or inhibition of the interaction of MSCs and cancer cells should be part of combinational therapies to improve prognosis. 

## 2. Results

### 2.1. Co-Culturing of MSCs and Cancer Cells Alter the Cytokine Secretion Profile

**Figure 1:**
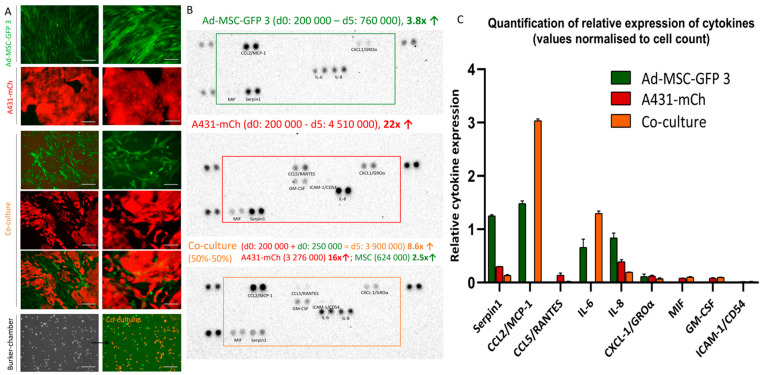
Representative image of cytokine secretion profile of 2D mono- and co-cultures of MSCs and A431 cancer cells. (**A**) representative images of mono- and co-cultures at day 0 and day 5. The number of MSCs and A431 cells were counted using Burker chambers under a fluorescence microscope. Scale bar on the left column is 200 µm and 100 µm on the right. The black arrow represents the change from brightfield to fluorescent imaging on the same field of view. (**B**) Cytokine secretion profile of MSCs, A431 cells, and co-cultures on the human cytokine profile membrane. (**C**) Relative cytokine secretion (normalized to cell numbers) of mono- and co-cultures.

Both MSCs and cancer cells are known to secrete a plethora of cytokines to communicate with and influence their microenvironment [5]; however, how secretion profiles change when the two cell types are cultured together has not been fully characterized. We investigated the secretion of 36 cytokines and chemokines in 2D mono- and co-cultures of MSCs and cancer cells. First, we established the growth kinetics of each cell line separately and in co-cultures (Figure 1A, Supplementary Figure S1). The Ad-MSC-GFP 3 cell population, initially seeded at 2 × 10^5^ cells, underwent a 3-fold increase in monocultures within a span of 5 days. In contrast, the A431-mCh cell population, also seeded at 2 × 10^5^ cells, exhibited a 20-fold increase under identical conditions. Co-cultures established with the same starting cell numbers of Ad-MSC-GFP and A431-mCh cells showed similar 2.5- and 16-fold increase, respectively, which allowed us to compare the cytokine profiles of mono- and co-cultures. 

Of the 36 studied cytokines/chemokines, only 9 were detectable in our model. The two cell lines showed similar secretion profiles. Both MSCs and A431 cells secreted MIF, SERPIN1, IL-8, and CXCL1/GRO⍺, while CCL2 and IL-6 were only found in MSC cultures, and CCL5 and GM-CSF were only detected in cancer cell supernatants. The cytokine profile of the co-cultured cells corresponded to the sum of the individual profiles with some subtle differences in most cases. SERPINE1, a cytokine known to be overexpressed and secreted by several tumor types to increase motility and tackle immune cell invasion [13], was strongly secreted by both cell types in monocultures; however, it became hardly detectable in co-cultures. The chemokine receptor CCL5 was described as overexpressed in tumors, contributing to microenvironment remodeling, cancer progression, DNA repair, and related processes [14]. In accordance with the literature, A431 cells secreted CCL5, but in co-cultures, CCL5 levels were hardly detectable. Although large amounts of IL-8 were observed in A431 supernatants and a substantial amount was secreted by MSCs, in the co-cultures, IL-8 levels were lower. Similarly, in the case of CXCL1/GRO⍺, co-culturing did not have any additional effect on secretion (Figure 1B,C). On the contrary, IL-6, one of the most investigated cytokines in cancer, was detected in MSCs and was virtually missing from A431 cultures; however, in co-cultures its secretion increased two-fold. Similarly, CCL2/MCP-1 was not secreted by A431 cells, but the relatively high levels detected in the MSC cultures were greatly increased when the two cell types were cultured together. While the similarity of the cytokines secreted by the MSCs and cancer cells is surprising, these increase in the secretion of two inflammatory cytokine, IL-6 and CCL2, suggests that even 120 h co-culturing with cancer cells could lead MSCs to establish a pro-inflammatory microenvironment.

### 2.2. Mesenchymal Stem Cells Do Not Influence Drug Tolerance in 2D Co-Cultures

**Figure 2:**
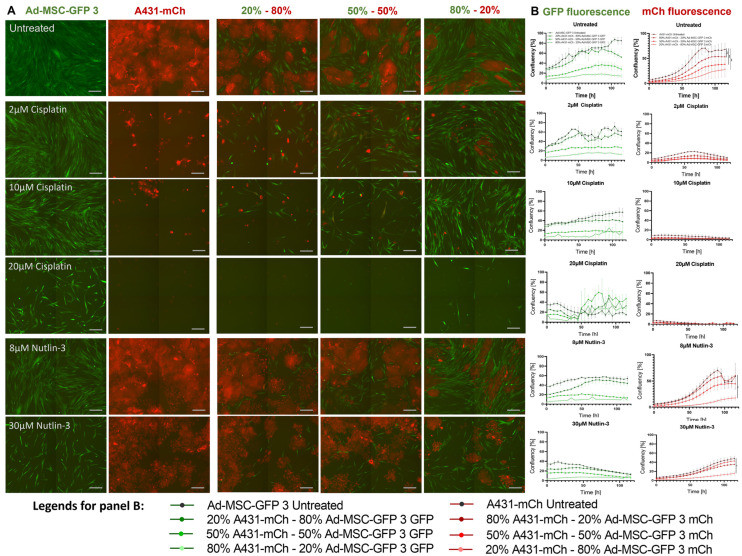
Cisplatin and nutlin-3 sensitivity of 2D mono- and co-cultures of MSCs and A431 cells. (**A**) Endpoint images of mono- and co-cultures of A431-mch and Ad-MSC-GFP 3 cells mixed in different ratios, following treatment with with 2 μM, 10 μM, and 20 μM cisplatin and 8 μM and 30 μM nutlin-3 for 5 days. Scale bar is 250 μm. Scale bar represents 250 µm. (**B**) Growth curves based on live cell imaging of mono- and co-cultures of MSCs (green) and cancer cells (red), imaged every 5 h for 120 h. *p* values and related significance levels were: *p* > 0.05 ns; *p* ≤ 0.05 *; *p* ≤ 0.01 **.

First, we tested whether Ad-MSC-GFP 3 have any effect on the growth rate of A431-mCh cells in co-cultures without any treatment. As shown in Supplementary Figure S2, there was no difference in the growth rate of A431-mCh mono- and co-cultures. Reports suggest that MSCs can influence the drug sensitivity of cancer cells, usually by increasing their drug tolerance [5]. Therefore, we tested cisplatin and nutlin-3 treatment on 2D co-cultures to investigate the effect of MSCs on the drug tolerability of A431 cells. The applied drug concentrations were based on the IC*_50_* values of cisplatin (2 µM (IC*_50_* of A431-mCH), 10 µM (IC*_50_* of Ad-MSC3-GFP 3)) and nutlin (8 µM (IC*_50_* of Ad-MSC-GFP 3) and 30 µM (IC*_50_* of A431). Using these compounds, we were able to investigate the susceptibility of both MSCs and cancer cells. However, 2D co-culturing altered neither cisplatin nor nutlin-3 sensitivity regardless of the ratio of MSCs and cancer cells (Figure 2A,B, Supplementary Video S1).

### 2.3. MSCs Provide a Scaffold for Cancer Cells in 3D Co-Culture Spheroids

**Figure 3:**
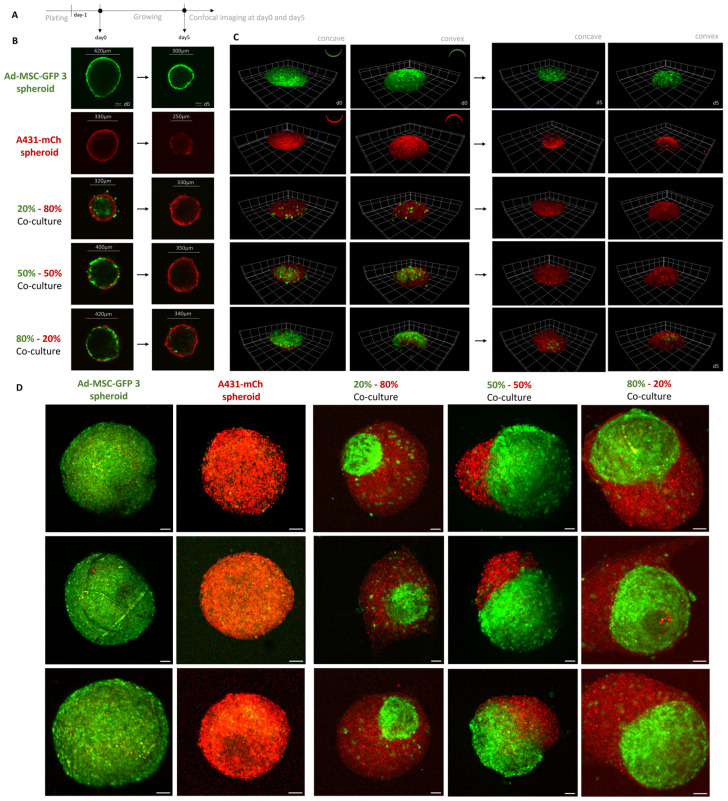
(**A**) Schematic experimental workflow of spheroid formation. (**B**,**C**) Confocal images showing the inner structure of 3D spheroids established from Ad-MSC-GFP 3 and A431-mCh cells. Cells mixed in different ratios were grown to allow for the formation of spheroids. Despite varying initial MSC:cancer cell ratios, the structure of the 3D co-cultures always shows the same pattern. Ad-MSC-GFP 3 cells create an inner scaffold for A431-mCh cells. Spheroids from A431-mCh cells die and collapse on their own, but if they are co-cultured with MSCs, they can survive on the surface of MSCs. X, Y and Z axis are the same size on all images. (**D**) Two-photon microscopy images of spheroids established using different cell ratios. In the first 3 columns Z-projection of the 3 replicates are shown. Scale bar 50 µm. The last column shows 3D reconstructed images of a single spheroid with the given cell ratios.

While in 2D co-cultures, MSCs and cancer cells did not show differences in sensitivity to cisplatin or nutlin-3, we established 3D spheroids from different ratios of MSCs and cancer cells to address the effect of MSCs on drug tolerance in a more relevant model (Figure 3A). Surprisingly, depending on the ratio of MSCs and A431 cells, spheroids emerging from the co-cultures seemed to separate into an MSC core and an outer layer formed from cancer cells. To further characterize the 3D structures, spheroids were imaged in several cell layer depths by confocal microscopy (Figure 3A) and two-photon microscopy (Figure 3B, Supplementary Figure S3). Both imaging techniques confirmed this pattern, indicating that MSCs provide a feeder core to cancer cells, similar to the fibroblast feeder layer used in embryonic and induced pluripotent stem cell culturing [15]. 

### 2.4. A Rare, Novel Double-Fluorescent Cell Population Arise in 2D Co-Cultures

**Figure 4:**
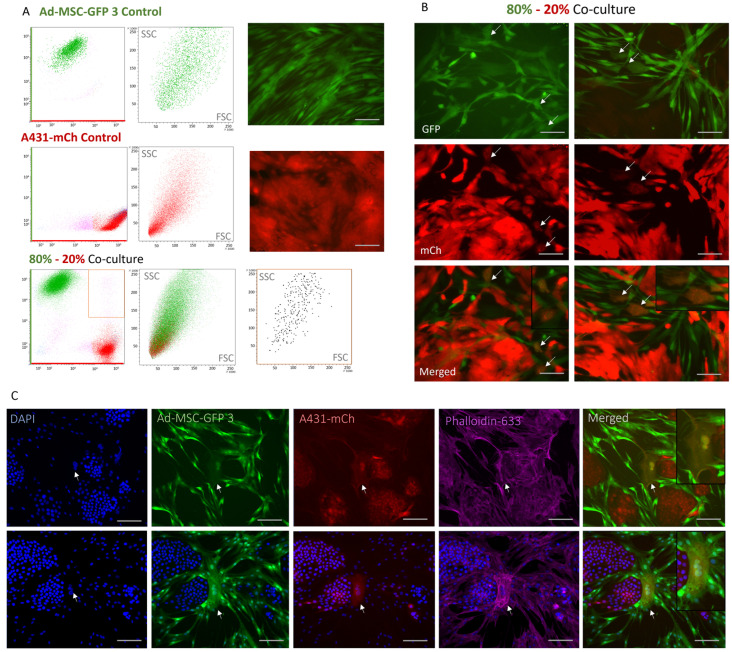
Analysis of GFP+/mCh+ double-positive cells. (**A**) Flow cytometry analysis of the composition of MSC, cancer cell and co-cultures, revealing that 0.8–1.1% of the co-culture shows double positivity. Green and red dots represent GFP- and mCh-positive cells, respectively. (**B**) Visualization of GFP+/mCh+ cells by fluorescent microscopy. White arrows point to double positive cells. Scale bar represent 70 μm. (**C**) F-actin staining with phalloidin (purple) of GFP- (green) and mCh-expressing (red) co-cultures and GFP+/mCh+ cells. Nuclei were visualized using DAPI (blue). Scale bars represent 200 μm. Double positive cells were magnified in the upper right corner of the merged images on both panel (**B**,**C**).

To probe the composition of the co-cultures, we performed flow cytometry studies. Analyzing the content of each spheroid verified the calculated cell ratios (Figure 4A). Interestingly, the analysis revealed a rare, newly emerged population of cells displaying both mCherry and GFP fluorescence (Figure 4B, Supplementary Figure S4) in accordance with Liu et al. [16], who recently reported similar findings. Imaging revealed that these cells consistently multinucleated and were significantly larger than A341 cells (Figure 4C), suggesting that the double-fluorescent population most probably originates from the MSC compartment of the co-cultures. Phalloidin staining of the double-fluorescent cells revealed not only changes in their morphology and size compared to the parental Ad-MSC and A431 cells, but also the formation of stress fibers in their cytoplasm, which could explain the altered cell shape (Figure 4B).

### 2.5. MSCs Increase Drug Tolerability of Cancer Cells in 3D Co-Cultures

**Figure 5:**
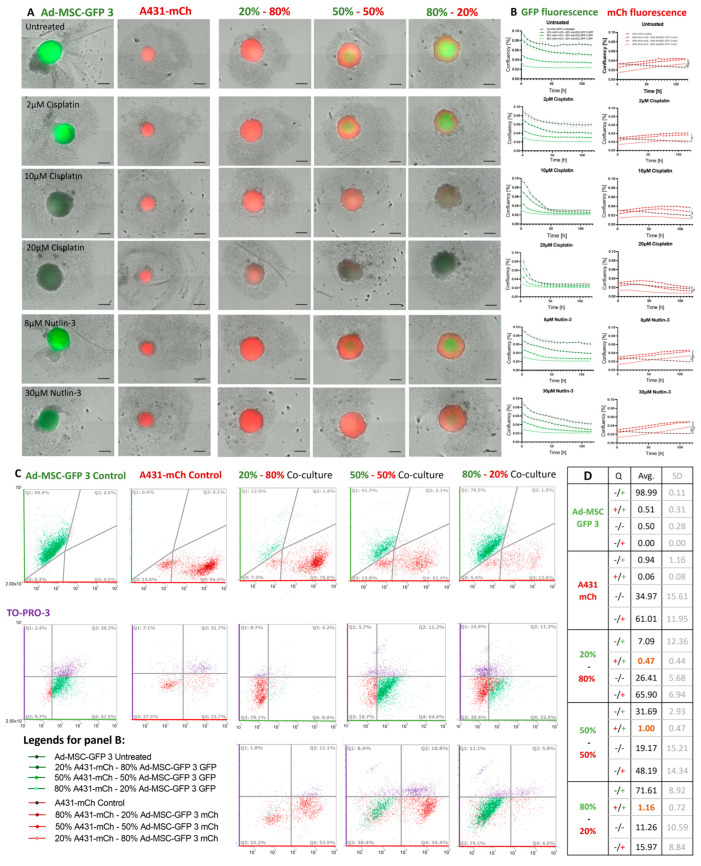
Cisplatin and nutlin-3 sensitivity of 3D Ad-MSC-GFP 3, A431-mCh and co-cultures. (**A**) Endpoint images of 3D co-cultured A431-mch and Ad-MSC-GFP 3 cells mixed in different ratios, treated with 2 μM, 10 μM, 20 μM cisplatin and 8 μM, 30 μM nutlin-3. Scale bars represent 250 μm. Cells were imaged every 5 h, creating a 120h record, and (**B**) growth curves were plotted by intensity analysis of GFP and mCherry fluorescence at each time point. (**C**) FACS analysis of mono- and co-cultured spheroids showing the number of GFP (Y axis, green)- and mCh (X axis, red)-expressing cells. Dead cells were stained with TO-PRO 3 (purple). (**D**) Quadrant statistics as determined by flow cytometry. P values and related significance levels were: *p* ≤ 0.05 *; *p* ≤ 0.01 **; *p* ≤ 0.001 ***.

While in 2D cultures MSCs did not change the drug sensitivity of cancer cells, we tested cisplatin and nutlin-3 sensitivity in the 3D spheroid co-cultures. In 3D mono-cultures both MSC and A431 spheroids become more compact over time, slightly shrinking in size. In contrast, cancer cells were able to grow under these conditions when co-cultured together with MSCs. This suggests that, despite their disadvantageous anchorage-free position, the presence of Ad-MSCs allowed them not just to avoid size reduction, but to even support proliferation (Figure 5A,B, Supplementary Video S2). For every experiment, spheroids, established using different cell ratios, with similar sizes were used to ensure reproducibility (Supplementary Figure S5).

Surprisingly, while in 2D cultures and in spheroid mono-cultures, A431 cells were killed by cisplatin at 2 μM concentration, cancer cells were able to grow at 10 μM in every tested cell ratio in 3D co-cultures. On the other hand, 10μM cisplatin was toxic to Ad-MSC spheroids (the same concentration did not affect the growth of Ad-MSC cells in 2D cultures), as shown by the reduced GFP fluorescence of the spheroids. Treatment with 20 μM cisplatin had a similar effect on Ad-MSC and also reduced the size of co-culture spheroids. Unexpectedly, A431-mCh cells were still alive in the 20–80% (Ad-MSC-GFP 3—A431-mCh) ratio co-cultures, indicating that MSCs alter the cancer cells’ drug sensitivity despite being killed or seriously injured. This observation is also supported by the results of the 30 μM nutlin-3 treatment. Nutlin-3 killed Ad-MSCs in all conditions, but the dying cells provided enough support for the A431 cells to grow considerably better than in similar mono-culture conditions. Remarkably, in the case of cisplatin, cancer cells survived even at higher concentrations despite being localized strictly on the outer layers of the spheroids. This indicates that the protective effect of MSCs is not based on physically sealing off A431 cells. To determine the number of dead cells in control and different spheroid conditions, we performed TO-PRO 3 staining. The cells that lost fluorescence are in the ‘Q3’ quadrant found to be TO-PRO 3 positive, showing that cells die in the spheroids (Figure 5C). Six spheroids were analyzed by flow cytometry and averaged as indicated in the table (Figure 5D).

## 3. Discussion

Cancer cells recruit many types of cells (immune cells, vascular endothelial cells, fibroblast/mesenchymal stem cells) to create the TME. Different tumor types have different tumor microenvironment compositions according to the size of the stroma part (MSCs/fibroblast) and the extension of the tumor part. In our work, we modeled the behavior of different proportions of mesenchymal stem cells in tumors and their influence on response to cisplatin and nutlin-3. In the clinical setting, calculation of tumor–stroma ratio (TSR) based on hematoxylin-eosin staining is a prognostic factor in colorectal cancer, breast cancer, or head-and-neck cancer and other solid malignant tumors. If the TSR percentage is above 50%, it indicates a high-stroma tumor, whereas if it falls below 50%, it indicates a low-stroma tumor type [17]. This value predicts treatment outcome in different types of cancer. A highly stromal triple-negative breast cancer has a worse outcome than a low-stroma non-triple-negative breast cancer [18].

To establish our co-culture system of MSCs and cancer cells, first we analyzed the effect of 2D co-culturing on cytokine secretion. While growing the two cell types together did not induce the secretion of different cytokines compared to mono-cultures, the secretion profile of the already detected molecules changed (Figure 1). Most importantly, the concentration of CCL2 and IL-6, which were secreted only by the MSCs, were doubled in the co-cultures suggesting that cancer cells can induce the secretion of some factors, like pro-inflammatory cytokines which could support their growth. On the other hand, the concentration of CCL5, secreted by A431 cells, decreased in co-cultures. Since CCL5 is an inflammatory cytokine which increases tumor growth, induces extracellular matrix remodeling, and enhances tumor cell migration [14], its decrease is in line with the remarkable anti-inflammatory effect of MSCs [19], suggesting that tumor-naïve MSCs can slow down cancer progression by altering the inflammatory TME. While IL-8 was secreted by both cells in mono-cultures, its levels decreased in co-cultures, but, in the contrary, the concentration of IL-6 increased showing a surprising shift in the IL-6-IL8 axis. These changes suggest that even short-term co-culturing can have a significant effect on the behavior of cancer cells and MSCs.

The observed changes may also explain how MSCs support the growth and survival of cancer cells with certain Ad-MSC:A431 ratios (Figure 5). There are significant discrepancies regarding the initial role of MSCs in early tumor development. Some studies claim that MSCs actively suppress cancer cells, while other works show the opposite. For example, the viability of MDA-MB-231 breast cancer cells decreased after being co-cultured with MSCs due to the inhibition of Notch signaling, an essential pathway for tumorigenesis [20]. Similarly, MSCs were reported to inhibit the growth of breast cancer cells by disrupting the Wnt pathway [21]. Additionally, MSCs regulate AKT activity in Kaposi’s sarcoma to limit tumor growth in vivo [22], increase mRNA expression of caspase 3, and p21 in tumor cells, and can stop cancer from spreading both in vitro and in vivo by inducing apoptosis and G0/G1 phase arrest. [23]. Conversely, MSCs can act in a pro-tumorigenic fashion by becoming carcinoma-associated MSCs [24] to induce angiogenesis [25], suppress the immune response and immune cell infiltration at the tumor site [26], increase motility and migration [27], and promote drug resistance [28]. The observations from our experiments indicate that MSCs facilitate the proliferation and survival of cancer cells only within specific ratios. This finding suggests that the role of MSCs in either promoting or inhibiting tumorigenesis may be influenced, at least partially, by the ratio of MSCs to cancer cells present in the TME. Furthermore, in 2D co-cultures, although there was some growth advantage observed, drug resistance did not develop in cancer cells. This suggests that mere physical proximity and altered cytokine secretion profiles may not suffice to protect the cells from cytotoxic drugs (Figure 2).

Surprisingly, the established 3D co-cultures showed a similar arrangement: MSCs providing a dense interior feeder core, while the A431 cells formed a mantle-like outer layer (Figure 3). Whether this structure is specific to these two cell lines remains to be further investigated. Reports suggest a similar 3D composition formed by human lung cancer cells and adipose tissue-derived mouse MSCs, using a thin chitosan–hyaluronan matrix-based substratum, called the “core-shell structure” [29]. On the other hand, according to a thought-provoking work on how cancer cells cannibalize MSCs and enter dormancy, the process may occur in the opposite direction: MSCs appear to envelop cancer cells in 3D spheroids [30]. Obviously, these phenotypes depend on the culture conditions and on the particular cell lines. Similarly to Han et al. [29], we used cell lines with epithelial phenotype (A431 and A549), while in the Bartosh et al. [30] paper MDA-MB-231, a well-known post-EMT cell line characterized by mesenchymal morphology was studied. 

We identified a rare, newly emergent subpopulation of cells which possessed the fluorescent properties of both Ad-MSC-GFP-3 and A431-mCh (Figure 4). Fluorescent microscopy revealed that these cells exhibited increased size compared to cancer cells and displayed a different morphology from MSCs. Most importantly, these cells had multiple nuclei. This phenomenon was described earlier by Liu et al. when squamous cell carcinoma (SCC) cells were co-cultured together with bone marrow MSCs [16]. Also, this these hybrid cells were reviewed before [31]. Similarly to our findings, the double-fluorescent cells characterized by Liu et al. [16] were rare (~1.6%) and displayed altered morphology along with the synthesis of stress fibers. However, while our study primarily identified mesenchymal morphology in this subpopulation, Liu et al. [16] predominantly observed epithelial morphology in the double-fluorescent population in what they termed MSC/SCC fused cells. Moreover, they suggested that these cells represent cancer cells that fused with MSCs, even showing tumorigenic potential. In our study, we propose that the double-fluorescent cells are more likely MSCs with incorporated cancer cells. Nonetheless, their true origin and role in tumor progression and drug resistance remain to be explored.

The most intriguing observations were made during the study of 3D spheroids. A431 spheroids were not able to grow under any circumstances when cultured alone; however, even the lowest number of additional MSCs were enough, not just to stabilize the cell number and avoid shrinking, but to support some growth (Figure 5). Moreover, co-culturing with MSCs increased drug tolerability of A431 cells and, while in 2D conditions, even 2 μM cisplatin inhibited proliferation and killed the cells, in the 3D co-cultures, treatment with 10 μM cisplatin was tolerable. This result is even more significant if we consider that cancer cells are on the outer layer (forming the “shell”) of the spheroid without any of the physical protection provided by the MSCs. Surprisingly, when we targeted the MSCs with nutlin-3, despite the diminishing inner core and cell death, MSCs were still able to support A431 cells. There are several ways that MSCs can promote drug resistance in cancer cells, mostly through the secretion of cytokines like IL-6, IL-7, and IL-8, for example, in head and neck carcinomas, where these molecules induce paclitaxel resistance [32]. In the 2D coculture system, IL-6 and IL-8 overexpression was not enough to increase drug resistance in cancer cells, suggesting that either another factor or the physical interaction between the cell types is missing. 

In a recently published work, the relationship between the quantity of the stromal compartment and prognosis was thoroughly analyzed across 16 solid tumor type [33]. The results questioned the common view that more reactive stroma in the tumor correlates with poorer prognosis. Our findings add to this argument, showing that the ratio, physical proximity, and secreted cytokines are all required for the MSCs to support the survival of cancer cells. In conclusion, our study confirms the contribution of MSCs to cancer drug resistance. Further research is necessary to clarify the contribution of all factors influencing this complex interaction.

## 4. Materials and Methods

### 4.1. Cell Culturing

Ad-MSC-GFP 3 [34] and A431-mCh cells were cultured in DMEM-F12 (Gibco, Carlsbad, CA, USA, 31331-028). DMEM-F12 was supplemented with 10 *v*/*v*% FBS (Gibco; 10270-106), 1% L-Glutamine (Lonza, Basel, Switzerland, BE17-605E), 0.1% 10 mg/mL gentamicin (Gibco, 15710064), and 1 ng/mL bFGF (Peprotech, London, UK). Lentiviral vectors expressing eGFP or mCherry genes under the control of the human EF1 promoter flanked by LTR sequences were generated using a second-generation packaging system for self-inactivating lentiviral vectors. The titer determination was performed by transduction of HEK293 cells followed by flow cytometry. When the percentage of infected cells is below 20%, the number of integrations is approximately equal to the number of transduced cells. Thus, the number of infectious virus particles per given volume can be determined as transduction units per mL (TU/mL). After the titer was known, target cells (A431, Ad-MSC-3) were transduced at an MOI of 1 (multiplicity of infection: the ratio of the number of transducing lentiviral particles to the number of cells), and then infected cells were sorted using a cell sorter until a completely homogeneous cell population was obtained based on fluorescent protein expression. Cells were cultured in T75 flasks at ~90% confluence; cultures were detached either with 0.1% (Ad-MSCs) or 0.2% (A431) trypsin (Sigma, St. Louis, MO, USA, T4799-100G diluted in PBS). The culture medium was changed every 3 days. Every experiment was repeated at least 3 times, and the standard deviation of all results was plotted on the graphs.

### 4.2. Establishing 3D Spheroid Mono- and Co-Cultures

A total of 10,000 cells from the Ad-MSC-GFP 3, A431-mCh cultures or their 80:20, 50:50, and 20:80 mixtures were seeded in 96-well U bottom microplates with cell-repellent surface (Greiner, Kremsmünster, Austria, 650970), and were allowed to self-aggregate for 24 h. Following the aggregation period, spheroids were thoroughly examined by fluorescent microscopy. Wells with multiple smaller spheroids instead of a single one were omitted from the study. The composition of the spheroids was examined using flow cytometry. Every experiment was repeated at least 3 times, and the standard deviation of all results was plotted on the graphs.

### 4.3. Cytokine Detection Assay

The Proteome Profiler Human Cytokine Array Kit (R&D systems, Minneapolis, MN, USA, ARY005B) was used to detect signaling molecules secreted by the cells. The investigated cytokines are listed in the Supplementary Table S1. For 2D mono-cultures, either 2 × 10^5^ Ad-MSC-GFP 3 or A431-mCh cells were seeded in a T25 tissue culture flask, while for the co-culture condition, 2 × 10^5^ Ad-MSC-GFP 3 and 2 × 10^5^ A431-mCh cells were seeded together. After overnight attachment, the medium was changed, and 72 h later, the medium was replaced with 2 mL serum-free DMEM-F12. Two days later, the supernatant was collected and centrifuged at 350 rcf for 5 min. For the detection of cytokines, samples were processed according to the manufacturer’s instructions. Briefly, the reagents were prepared in advance and 2 mL of Array Buffer 4 was pipetted into the chambers of the kit’s multi-dish reaction plate with the Human Cytokine Array nitrocellulose membranes spotted with 36 different antibodies. The samples were incubated at room temperature for 1 h. Meanwhile, 500 μL Array Buffer 4, 200 μL Array Buffer 5, 800 μL supernatant, and 15 μL Human Cytokine Array Detection Antibody Cocktail was mixed gently and incubated for 1 h. Array Buffer 4 was removed from the membranes, replaced by the sample-antibody mixture (1.515 μL), and incubated overnight at 4 °C. Membranes were placed into a 50 mL falcon tube and rinsed with 1× Wash Buffer on rocking platform shaker three times. Streptavidin-HRP (as a positive control) was diluted 2000-fold in Array Buffer 5. The membrane and the diluted Streptavidin-HRP solution were placed into the washed membrane container and incubated for 30 min at room temperature on a platform shaker. The membrane was washed again with 1× Wash Buffer 3 times for 10 min. A 1 mL quantity of Chemi Reagent Mix was prepared, and after 1 min, the membranes were washed, incubated for 2 min, and placed in a plastic sheet container, and the air bubbles were removed. Membranes were imaged with the ChemiDoc™ MP (Bio-Rad, Hercules, CA, USA) Imaging System. Every experiment was repeated at least 3 times, and the standard deviation of all results was plotted on the graphs.

### 4.4. Drug Treatment

Either 24 h after cell seeding in 96-well plates (5000 cells/well) or after spheroid aggregation, cultures were treated with 2 μM, 10 μM, and 20 μM cisplatin or 8 μM and 30 μM of nutlin-3, respectively. Viability was assessed by continuous monitoring of the GFP and mCherry fluorescence of the cultures for 120 h, using the JuLI Stage fluorescent live-cell imaging system (NanoEntek, Seoul, Republic of Korea). Cisplatin (Cat. No: Vnr461201) was obtained from Accord Healthcare, UK; nutlin-3 (Cat. No: 3984/10) was obtained from Tocris Bioscience (Bristol, UK). 

### 4.5. Flow Cytometry Analysis

Cells were seeded in a T75 flask at a ratio of 1:4 (A431-mCh:Ad-MSC-GFP 3) and allowed to attach overnight. The next day, the medium was replaced with 12 mL of fresh DMEM-F12. After 120 h, cultures were trypsinized, and single cells were analyzed by flow cytometry with FACS Aria III. For spheroids, the culture medium was removed from the 96-well plate and was washed gently with PBS, removing as much liquid as possible. Then, spheroids were digested in 70 μL 0.2% trypsin for 15 min, mechanically dissociated with a pipette, and digested for another 10 min. The single-cell solution was diluted with culture media to stop the enzymatic reaction. The cells were washed gently with PBS before flow cytometry analysis. GFP+/mCherry+ double-fluorescent cells were sorted with a FACS Aria III (GFP: 488 laser, 530/30; mCh: 561 laser, 610/20). Spheroids were digested with 0.2% trypsin for 30 min to achieve a single-cell suspension. Cells were labeled with TO-PRO 3. Attune Nxt flow cytometer was used to analyze the cells (GFP: 488 laser, BL1 530/30, mCh: 561 laser, YL2 620/15; TO-PRO 3 638 laser, RL1 670/14).

### 4.6. Live-Cell, Confocal and Two-Photon Imaging of Cell and Spheroid Cultures

Cells were imaged with an Olympus IX51 microscope with a connected SPOT RT3 camera, time-lapse videos were recorded with the JuLI Stage fluorescence live-cell imaging system, and the images were edited with JuLI EDIT. JuLI Stat was used to analyze the growth kinetics of cells based on fluorescence images. Briefly, the area occupied by either green (GFP) or red (mCherry) pixels were compared to the whole area on all images (24 image/120 h experiment) and was expressed as confluency (%). Confocal microscopy was performed using a Zeiss LSM-710 microscope (Carl Zeiss, Jena, Germany) with the same settings for all samples. For two-photon microscopy, the spheroids were transferred to 30 mm standing cell culture inserts (Millicell PICM03050, Millipore, Burlington, MA, USA) in no more than 10 µL culture media and were imaged using a two-photon microscope (Femto2D, Femtonics, Budapest, Hungary) equipped with a 4× dry objective (Olympus, Tokyo, Japan, PLN 4X, N.A. 0.10). GFP and mCherry were excited at 920 nm with a femtosecond laser source (Toptica FF Ultra 920, Toptica Photonics AG, Grafelfing, Germany). The emitted GFP and mCherry fluorescence were monitored at 475–575 and 600–700 nm, respectively. Image reconstruction was carried out using Fiji v2.15.

### 4.7. Analysis of Spheroid Size and Growth Rate

Multi-channel images acquired from the spheroid cultures using the JuLI Stage fluorescent live-cell imaging system underwent batch quantification using CellProfiler v4.2.6 [11]. To ensure comprehensive coverage of the well surfaces and to exclude potential image artifacts near the edges, four neighboring locations were selected for imaging in each well. In all instances, fluorescent signals were recorded from two channels (GFP and mCherry).

Initially, per-channel images captured from the same well were combined (tiled) into a larger image without any rescaling or equalization, preserving the originally acquired fluorescence signal intensities. Subsequently, the obtained grayscale images were quantified using CellProfiler’s intensity measurement module, resulting in mean signal intensities for each combined image. The computed mean signal intensity (the sum of all pixel intensities divided by the number of pixels in the image) ranged from 0.0 for an image devoid of any signal to 1.0 for an image with the highest possible intensities for each pixel throughout the image. Processing of signals from both fluorescent channels was conducted independently.

### 4.8. Statistical Analysis

Statistical analyses were performed using the GraphPad Prism version 8.0.0 for Windows, GraphPad Software (San Diego, CA, USA). Two-tailed Student’s *t*-test was used to compare the growth kinetics of different 2D cultures, and 3D mono- and co-culture spheroids with or without treatment. The calculated p values was included on the given figures.

## Data Availability

Data is contained within the article and Supplementary Material.

## Supplementary Materials

The following supporting information can be downloaded at: https://www.mdpi.com/article/10.3390/ijms25084515/s1.
