# Supplementary material for: Mesenchymal Stem Cells Increase Drug Tolerance of A431 Cells Only in 3D Spheroids, Not in 2D Co-Cultures
Source: Int J Mol Sci. 2024 Apr 20;25(8):4515. doi: 10.3390/ijms25084515 (PMC11049889; doi:10.3390/ijms25084515)
Supplement: Supplementary file 1 [file ijms-25-04515-s001.zip › Supplement_Vajda et al_rev.pdf]

# Supplementary Material to Mesenchymal Stem Cells increase drug tolerance of A431 cells only in 3D spheroids, but not in 2D co-cultures by Vajda et al.

## Cytokine secretion assay, list of detectable cytokines, chemokines

The Proteome Profiler Human Cytokine Array Kit (ARY005) detects 36 human cytokines, chemokines, and acute phase proteins simultaneously (Table 1.). This nitrocellulose membrane-based antibody array is designed for detection the relative level of human cytokines and chemokines. A concentrated cell culture supernatant was mixed with a biotinylated antibodies and immobilized on the membrane.

**Table S1.: List of the simultaneously detectable cytokines, chemokines in one sample.** Names of the 36 cytokines in alphabetical order.

| | | |
|---|---|---|
| C5a | IL-4 | IL-27 |
| CD40 Ligand | IL-5 | IL-32 alpha |
| G-CSF | IL-6 | CXCL10/IP-10 |
| GM-CSF | IL-8 | CXCL11/I-TAC |
| CXCL1/GRO alpha | IL-10 | CCL2/MCP-1 |
| CCL1/I-309 | IL-12 p70 | MIF |
| ICAM-1 | IL-13 | MIP-1 alpha/MIP-1 beta |
| IFN-gamma | IL-16 | CCL5/RANTES |
| IL-1 alpha | IL-17 | CXCL12/SDF-1 |
| IL-1 beta | IL-17E | Serpin E1/PAI-1 |
| IL-1ra | IL-18 | TNF-alpha |
| IL-2 | IL-21 | TREM-1 |

Cell growth was also monitored by cell counting at day 5 during the cytokine assay. In each case, the initial cell count was 200 000 cells from each cell line, which increased 20x for tumour cells and 2.5x times for MSCs (Supplementary Figure 1.).

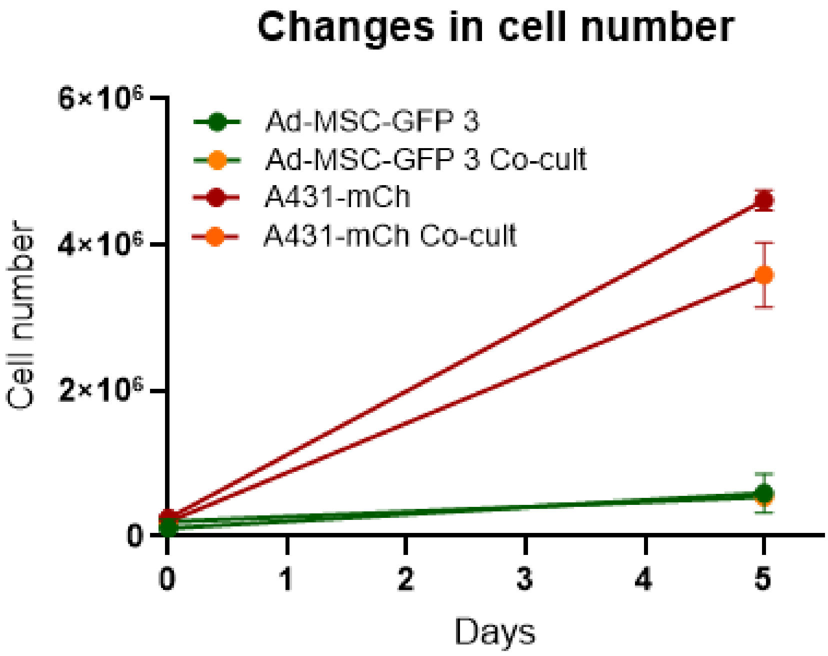


**Supplementary Figure S1.** Cell growth kinetics in 2D mono-and co-cultured Ad-MSC-GFP 3 and A431-mCh cells.

**Mesenchymal Stem Cells doesn't influence the growth of A431 cells in 2D co-cultures**

Using a wider range of different A431-mch and Ad-MSC-GFP 3 cell ratios showed that there is no significant support for A431's growth in co-cultures compared to mono-cultures (Supplementary Figure 2A, B).

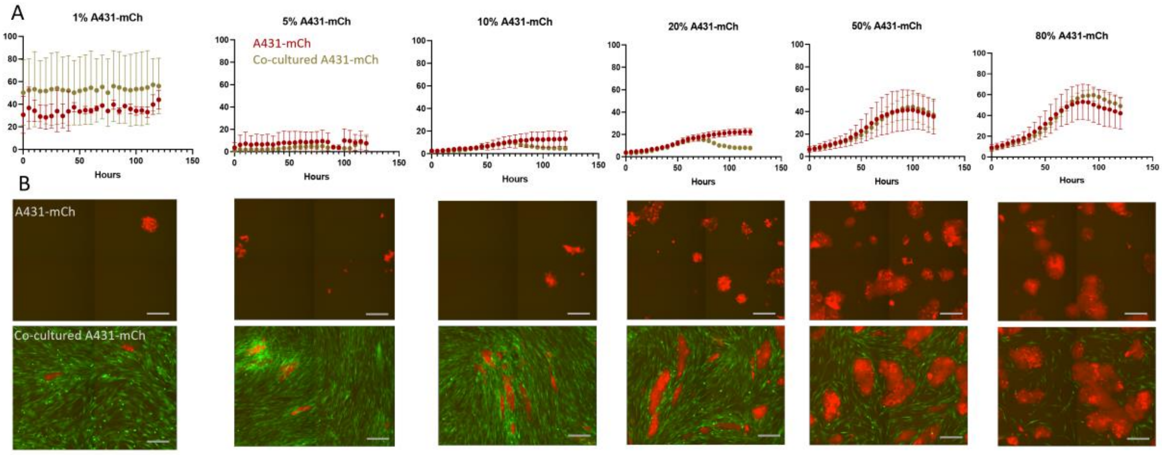


**Supplementary Figure S2. Tumor cell growth at different MSC: cancer cell ratios (99%-1%, 95%-5%, 90%-10%, 80%-20%, 50%-50%, 20%-80%).** (A) Growth kinetics calculation was based on mCherry fluorescence and (B) morphology of mono-and co-cultured A431-mCh cells during a 120h long observational period. Scale bars represent 250 μm.

**Two-photon microscopy imaging of 3D mono- and co-culture spheroids**

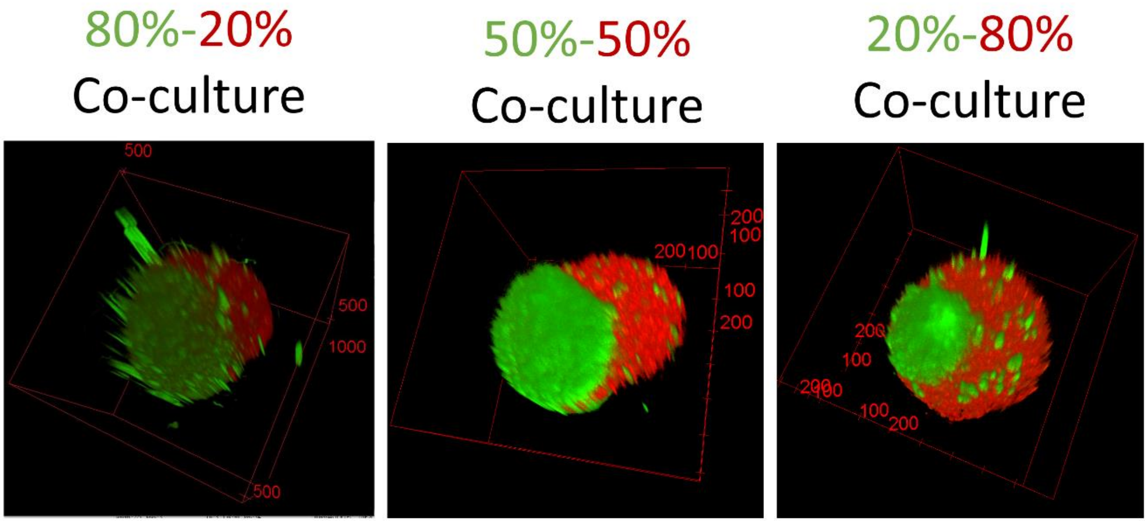


**Supplementary Figure S3. Two-photon microscopy images of spheroids established using different cell ratios.** The figures show the 3D reconstructed images of a single spheroid with the given cell ratios.

**The appearance of the rare, double-fluorescent cell population is not depending on the expression of a given fluorescent protein**

$GFP^+/mCh^+$ double-fluorescent cells could be a result of a fluorescent protein-related issue, like mCherry is more stable in culture media after cell death, or that is more likely to phagocytosed by other cells than GFP. To investigate whether the cell line specific expression of certain fluorescent proteins is influencing the double-fluorescent population we also performed inverse expression of GFP and mCherry in MSCs and A431. After co-culturing Ad-MSC-mCh 1 and A431-GFP cells, in 80%-20% ratio FACS analysis strengthen the previous finding that double-positive cells are observed in co-culture. A431-GFP cells and Ad-MSC-mCh 1 cells were tested as controls in GFP and mCherry channels. According to the GFP-mCh panels in 80%-20% co-culture in 0.8% appeared $GFP^+/mCh^+$ cells (Supplementary Figure 4A). Fluorescence images show double-positive cells in GFP and mCherry channels (Supplementary Figure 4B).

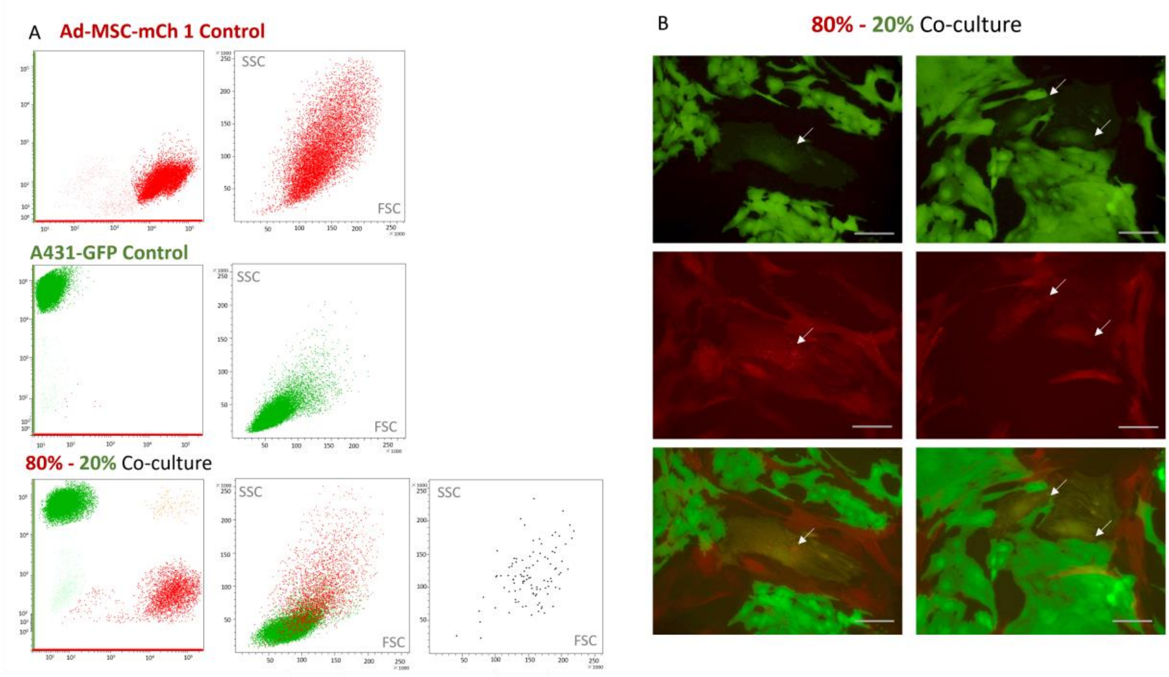


**Supplementary Figure S4. Co-culturing Ad-MSC-mCherry 1 and A431-GFP cells results in the appearance of a similar double-fluorescent population as in the experiments with opposite fluorescent tags.** (A) Flow cytometry analysis of the composition of MSCs, cancer cells and co-cultures. (B) Validation of $GFP^+/mCh^+$ cells using fluorescent microscopy. Scale bars represents 250 µm.

**Average diameter of mono-and co-cultured spheroids**

The diameter of the spheroids was measured in -µm- on day 1 and the results were summarised in Supplementary Figure 5. Spheroid with the largest mass and diameter was given by mono-cultured Ad-MSC-GFP 3 cells and A431-mCh cells formed the smallest spheroid. The higher the proportion of MSCs in the co-culture, the larger

spheroids were formed. The proportion of MSCs influence the diameter of the spheroids, which can be explained by the difference in size and shape of the two cell types. Spheroids established from 10 000 cells in each cases, varied in diameter from 350 to 600µm (Supplementary Figure 5.).

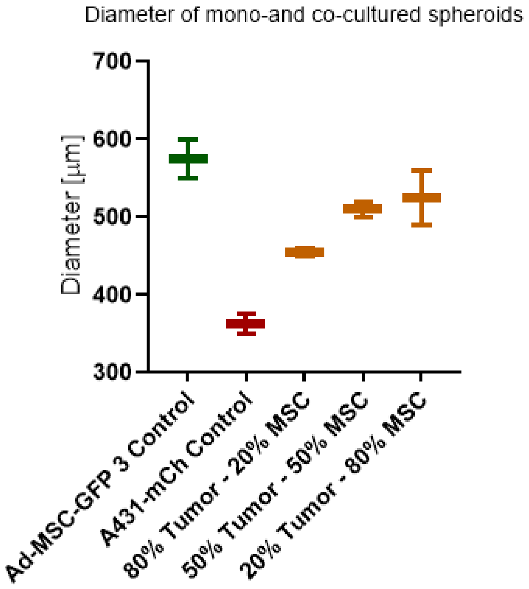


**Supplementary Figure S5.:** Diameter of mono-and co-cultured Ad-MSC-gfp 3 and A431-mCh spheroids.
